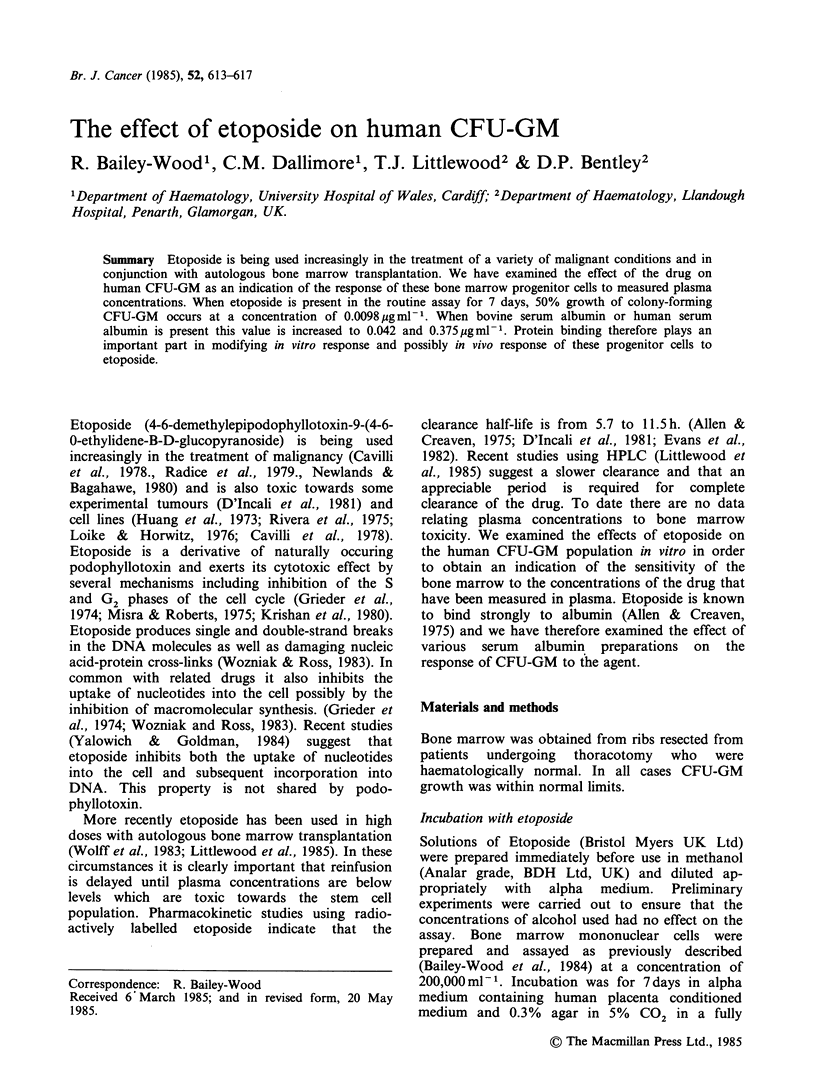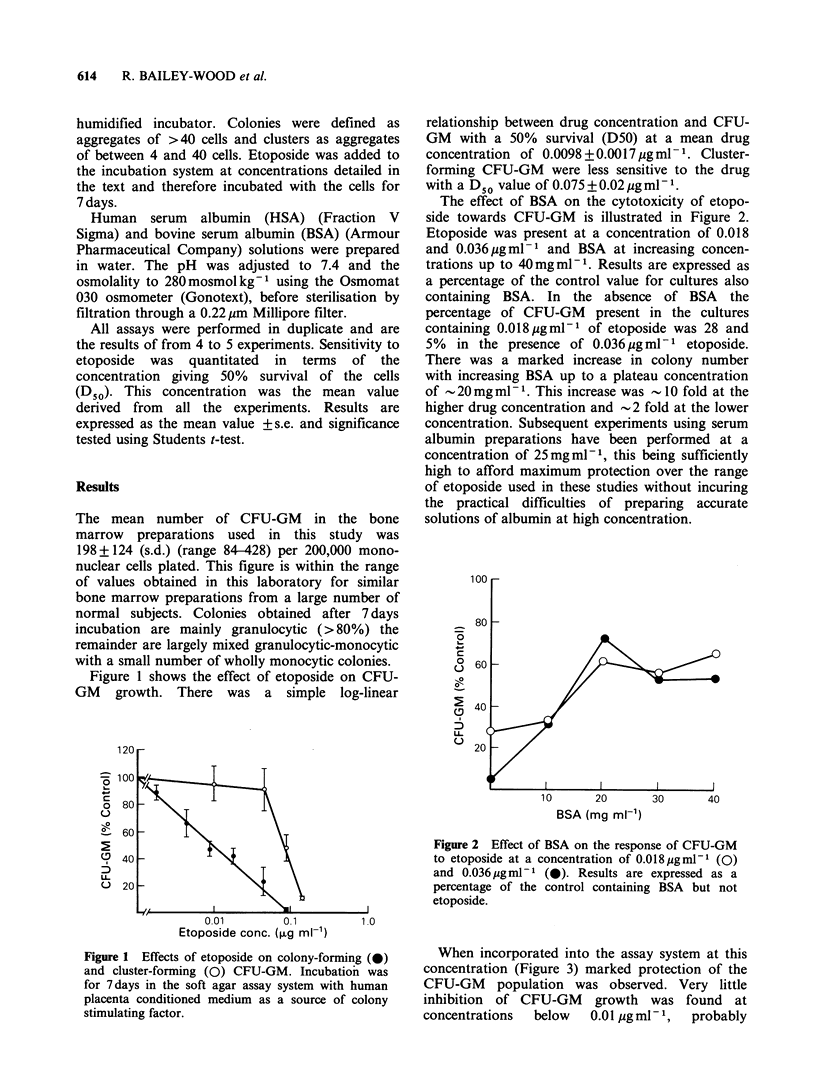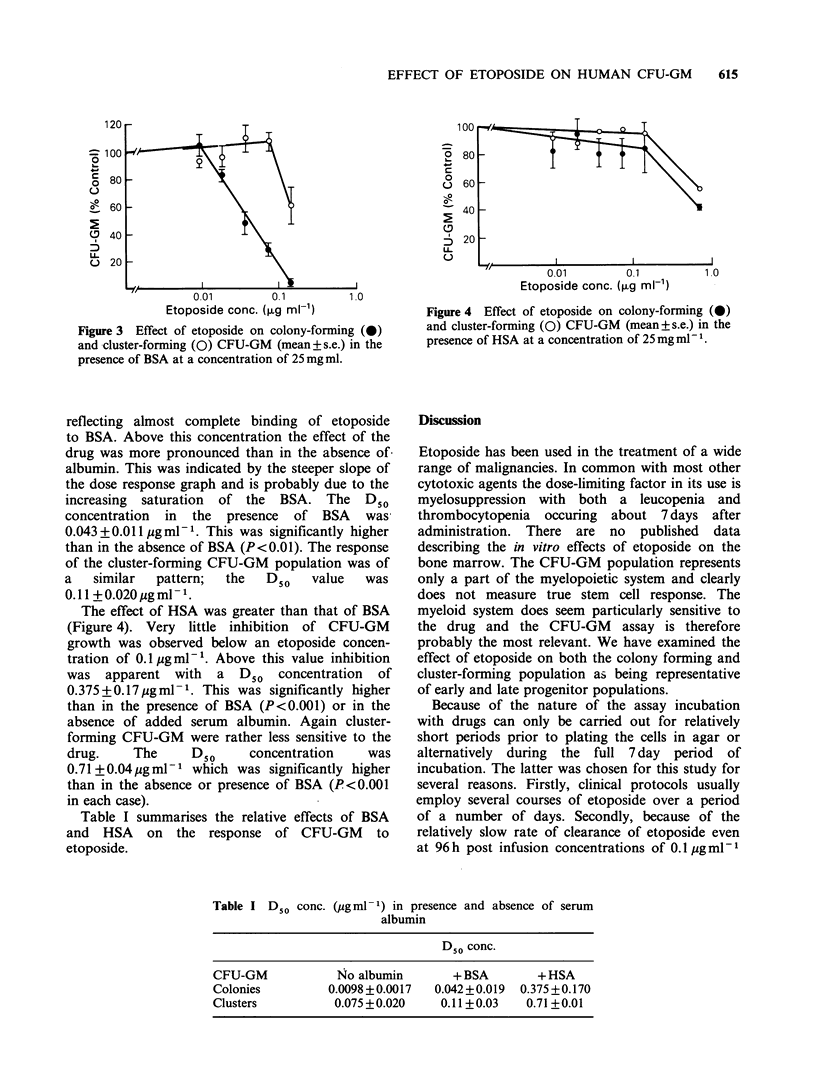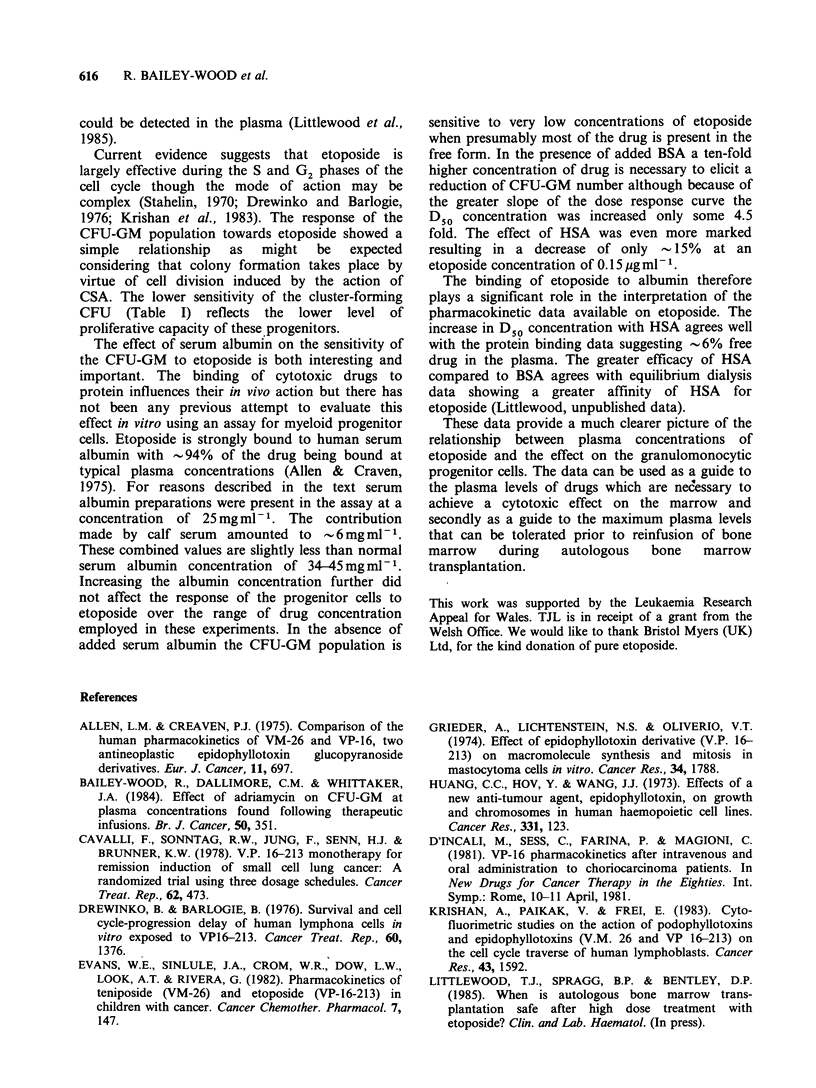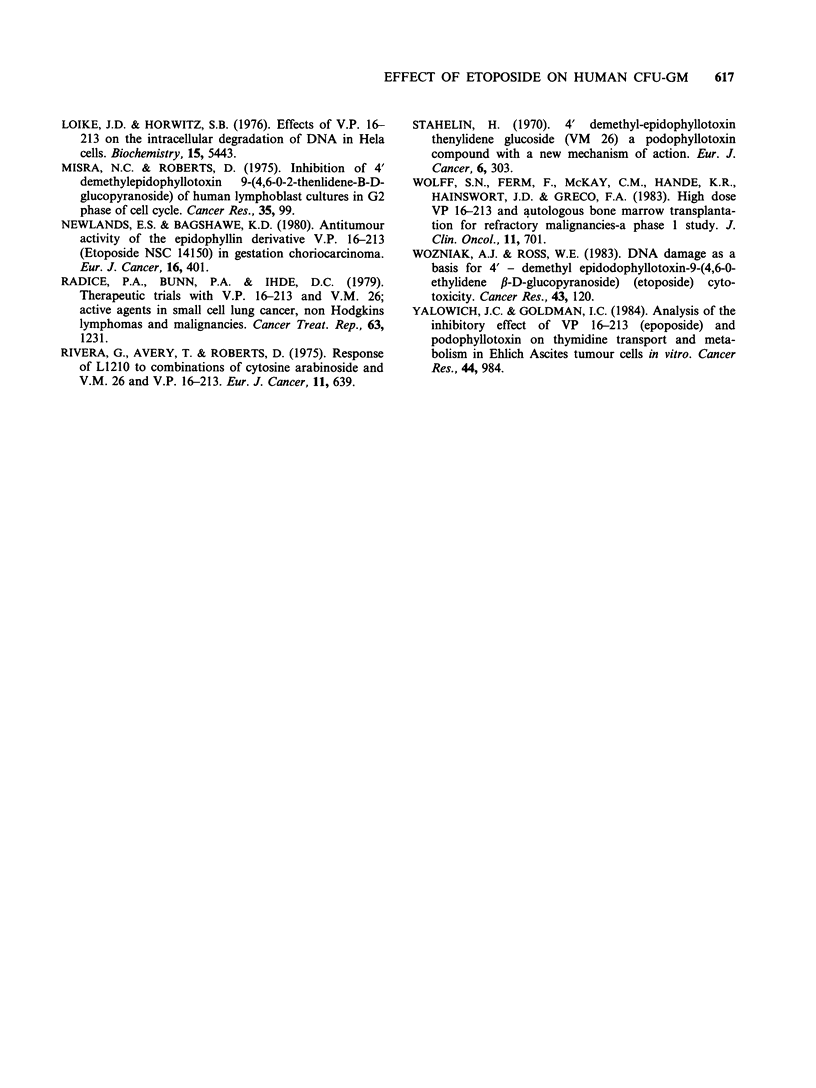# The effect of etoposide on human CFU-GM.

**DOI:** 10.1038/bjc.1985.234

**Published:** 1985-10

**Authors:** R. Bailey-Wood, C. M. Dallimore, T. J. Littlewood, D. P. Bentley

## Abstract

Etoposide is being used increasingly in the treatment of a variety of malignant conditions and in conjunction with autologous bone marrow transplantation. We have examined the effect of the drug on human CFU-GM as an indication of the response of these bone marrow progenitor cells to measured plasma concentrations. When etoposide is present in the routine assay for 7 days, 50% growth of colony-forming CFU-GM occurs at a concentration of 0.0098 micrograms ml-1. When bovine serum albumin or human serum albumin is present this value is increased to 0.042 and 0.375 micrograms ml-1. Protein binding therefore plays an important part in modifying in vitro response and possibly in vivo response of these progenitor cells to etoposide.


*Br. J. Cancer* (1985), **52**, 613–617

### The effect of etoposide on human CFU-GM

R. Bailey-Wood[1], C.M. Dallimore[1], T.J. Littlewood[2] & D.P. Bentley[2]

[1]*Department of Haematology, University Hospital of Wales, Cardiff;* [2]*Department of Haematology, Llandough Hospital, Penarth, Glamorgan, UK.*

**Summary** Etoposide is being used increasingly in the treatment of a variety of malignant conditions and in conjunction with autologous bone marrow transplantation. We have examined the effect of the drug on human CFU-GM as an indication of the response of these bone marrow progenitor cells to measured plasma concentrations. When etoposide is present in the routine assay for 7 days, 50% growth of colony-forming CFU-GM occurs at a concentration of $0.0098\,\mu g\,ml^{-1}$. When bovine serum albumin or human serum albumin is present this value is increased to 0.042 and $0.375\,\mu g\,ml^{-1}$. Protein binding therefore plays an important part in modifying *in vitro* response and possibly *in vivo* response of these progenitor cells to etoposide.

Etoposide (4-6-demethylepipodophyllotoxin-9-(4-6-0-ethylidene-B-D-glucopyranoside) is being used increasingly in the treatment of malignancy (Cavilli *et al.*, 1978., Radice *et al.*, 1979., Newlands & Bagahawe, 1980) and is also toxic towards some experimental tumours (D'Incali *et al.*, 1981) and cell lines (Huang *et al.*, 1973; Rivera *et al.*, 1975; Loike & Horwitz, 1976; Cavilli *et al.*, 1978). Etoposide is a derivative of naturally occuring podophyllotoxin and exerts its cytotoxic effect by several mechanisms including inhibition of the S and $G_2$ phases of the cell cycle (Grieder *et al.*, 1974; Misra & Roberts, 1975; Krishan *et al.*, 1980). Etoposide produces single and double-strand breaks in the DNA molecules as well as damaging nucleic acid-protein cross-links (Wozniak & Ross, 1983). In common with related drugs it also inhibits the uptake of nucleotides into the cell possibly by the inhibition of macromolecular synthesis. (Grieder *et al.*, 1974; Wozniak and Ross, 1983). Recent studies (Yalowich & Goldman, 1984) suggest that etoposide inhibits both the uptake of nucleotides into the cell and subsequent incorporation into DNA. This property is not shared by podophyllotoxin.

More recently etoposide has been used in high doses with autologous bone marrow transplantation (Wolff *et al.*, 1983; Littlewood *et al.*, 1985). In these circumstances it is clearly important that reinfusion is delayed until plasma concentrations are below levels which are toxic towards the stem cell population. Pharmacokinetic studies using radio-actively labelled etoposide indicate that the clearance half-life is from 5.7 to 11.5h. (Allen & Creaven, 1975; D'Incali *et al.*, 1981; Evans *et al.*, 1982). Recent studies using HPLC (Littlewood *et al.*, 1985) suggest a slower clearance and that an appreciable period is required for complete clearance of the drug. To date there are no data relating plasma concentrations to bone marrow toxicity. We examined the effects of etoposide on the human CFU-GM population *in vitro* in order to obtain an indication of the sensitivity of the bone marrow to the concentrations of the drug that have been measured in plasma. Etoposide is known to bind strongly to albumin (Allen & Creaven, 1975) and we have therefore examined the effect of various serum albumin preparations on the response of CFU-GM to the agent.

### Materials and methods

Bone marrow was obtained from ribs resected from patients undergoing thoracotomy who were haematologically normal. In all cases CFU-GM growth was within normal limits.

### *Incubation with etoposide*

Solutions of Etoposide (Bristol Myers UK Ltd) were prepared immediately before use in methanol (Analar grade, BDH Ltd, UK) and diluted appropriately with alpha medium. Preliminary experiments were carried out to ensure that the concentrations of alcohol used had no effect on the assay. Bone marrow mononuclear cells were prepared and assayed as previously described (Bailey-Wood *et al.*, 1984) at a concentration of $200{,}000\,ml^{-1}$. Incubation was for 7days in alpha medium containing human placenta conditioned medium and 0.3% agar in 5% $CO_2$ in a fully

Correspondence: R. Bailey-Wood
Received 6 March 1985; and in revised form, 20 May 1985.

© The Macmillan Press Ltd., 1985

humidified incubator. Colonies were defined as aggregates of >40 cells and clusters as aggregates of between 4 and 40 cells. Etoposide was added to the incubation system at concentrations detailed in the text and therefore incubated with the cells for 7 days.

Human serum albumin (HSA) (Fraction V Sigma) and bovine serum albumin (BSA) (Armour Pharmaceutical Company) solutions were prepared in water. The pH was adjusted to 7.4 and the osmolality to 280 mosmol kg$^{-1}$ using the Osmomat 030 osmometer (Gonotext), before sterilisation by filtration through a 0.22 $\mu$m Millipore filter.

All assays were performed in duplicate and are the results of from 4 to 5 experiments. Sensitivity to etoposide was quantitated in terms of the concentration giving 50% survival of the cells ($D_{50}$). This concentration was the mean value derived from all the experiments. Results are expressed as the mean value $\pm$s.e. and significance tested using Students *t*-test.

### Results

The mean number of CFU-GM in the bone marrow preparations used in this study was 198$\pm$124 (s.d.) (range 84–428) per 200,000 mononuclear cells plated. This figure is within the range of values obtained in this laboratory for similar bone marrow preparations from a large number of normal subjects. Colonies obtained after 7 days incubation are mainly granulocytic (>80%) the remainder are largely mixed granulocytic-monocytic with a small number of wholly monocytic colonies.

Figure 1 shows the effect of etoposide on CFU-GM growth. There was a simple log-linear relationship between drug concentration and CFU-GM with a 50% survival (D50) at a mean drug concentration of 0.0098$\pm$0.0017 $\mu$g ml$^{-1}$. Cluster-forming CFU-GM were less sensitive to the drug with a $D_{50}$ value of 0.075$\pm$0.02 $\mu$g ml$^{-1}$.

**Figure 1** Effects of etoposide on colony-forming (●) and cluster-forming (○) CFU-GM. Incubation was for 7 days in the soft agar assay system with human placenta conditioned medium as a source of colony stimulating factor.

The effect of BSA on the cytotoxicity of etoposide towards CFU-GM is illustrated in Figure 2. Etoposide was present at a concentration of 0.018 and 0.036 $\mu$g ml$^{-1}$ and BSA at increasing concentrations up to 40 mg ml$^{-1}$. Results are expressed as a percentage of the control value for cultures also containing BSA. In the absence of BSA the percentage of CFU-GM present in the cultures containing 0.018 $\mu$g ml$^{-1}$ of etoposide was 28 and 5% in the presence of 0.036 $\mu$g ml$^{-1}$ etoposide. There was a marked increase in colony number with increasing BSA up to a plateau concentration of ~20 mg ml$^{-1}$. This increase was ~10 fold at the higher drug concentration and ~2 fold at the lower concentration. Subsequent experiments using serum albumin preparations have been performed at a concentration of 25 mg ml$^{-1}$, this being sufficiently high to afford maximum protection over the range of etoposide used in these studies without incuring the practical difficulties of preparing accurate solutions of albumin at high concentration.

**Figure 2** Effect of BSA on the response of CFU-GM to etoposide at a concentration of 0.018 $\mu$g ml$^{-1}$ (○) and 0.036 $\mu$g ml$^{-1}$ (●). Results are expressed as a percentage of the control containing BSA but not etoposide.

When incorporated into the assay system at this concentration (Figure 3) marked protection of the CFU-GM population was observed. Very little inhibition of CFU-GM growth was found at concentrations below 0.01 $\mu$g ml$^{-1}$, probably

**Figure 3** Effect of etoposide on colony-forming (●) and cluster-forming (○) CFU-GM (mean ± s.e.) in the presence of BSA at a concentration of 25 mg ml.

**Figure 4** Effect of etoposide on colony-forming (●) and cluster-forming (○) CFU-GM (mean ± s.e.) in the presence of HSA at a concentration of 25 mg ml$^{-1}$.

reflecting almost complete binding of etoposide to BSA. Above this concentration the effect of the drug was more pronounced than in the absence of albumin. This was indicated by the steeper slope of the dose response graph and is probably due to the increasing saturation of the BSA. The $D_{50}$ concentration in the presence of BSA was $0.043 \pm 0.011\,\mu g\,ml^{-1}$. This was significantly higher than in the absence of BSA ($P < 0.01$). The response of the cluster-forming CFU-GM population was of a similar pattern; the $D_{50}$ value was $0.11 \pm 0.020\,\mu g\,ml^{-1}$.

The effect of HSA was greater than that of BSA (Figure 4). Very little inhibition of CFU-GM growth was observed below an etoposide concentration of $0.1\,\mu g\,ml^{-1}$. Above this value inhibition was apparent with a $D_{50}$ concentration of $0.375 \pm 0.17\,\mu g\,ml^{-1}$. This was significantly higher than in the presence of BSA ($P < 0.001$) or in the absence of added serum albumin. Again cluster-forming CFU-GM were rather less sensitive to the drug. The $D_{50}$ concentration was $0.71 \pm 0.04\,\mu g\,ml^{-1}$ which was significantly higher than in the absence or presence of BSA ($P < 0.001$ in each case).

Table I summarises the relative effects of BSA and HSA on the response of CFU-GM to etoposide.

### Discussion

Etoposide has been used in the treatment of a wide range of malignancies. In common with most other cytotoxic agents the dose-limiting factor in its use is myelosuppression with both a leucopenia and thrombocytopenia occuring about 7 days after administration. There are no published data describing the *in vitro* effects of etoposide on the bone marrow. The CFU-GM population represents only a part of the myelopoietic system and clearly does not measure true stem cell response. The myeloid system does seem particularly sensitive to the drug and the CFU-GM assay is therefore probably the most relevant. We have examined the effect of etoposide on both the colony forming and cluster-forming population as being representative of early and late progenitor populations.

Because of the nature of the assay incubation with drugs can only be carried out for relatively short periods prior to plating the cells in agar or alternatively during the full 7 day period of incubation. The latter was chosen for this study for several reasons. Firstly, clinical protocols usually employ several courses of etoposide over a period of a number of days. Secondly, because of the relatively slow rate of clearance of etoposide even at 96 h post infusion concentrations of $0.1\,\mu g\,ml^{-1}$

**Table I** $D_{50}$ conc. ($\mu g\,ml^{-1}$) in presence and absence of serum albumin

| | $D_{50}$ conc. | | |
|---|---|---|---|
| CFU-GM | No albumin | +BSA | +HSA |
| Colonies | $0.0098 \pm 0.0017$ | $0.042 \pm 0.019$ | $0.375 \pm 0.170$ |
| Clusters | $0.075 \pm 0.020$ | $0.11 \pm 0.03$ | $0.71 \pm 0.01$ |

could be detected in the plasma (Littlewood *et al.*, 1985).

Current evidence suggests that etoposide is largely effective during the S and $G_2$ phases of the cell cycle though the mode of action may be complex (Stahelin, 1970; Drewinko and Barlogie, 1976; Krishan *et al.*, 1983). The response of the CFU-GM population towards etoposide showed a simple relationship as might be expected considering that colony formation takes place by virtue of cell division induced by the action of CSA. The lower sensitivity of the cluster-forming CFU (Table I) reflects the lower level of proliferative capacity of these progenitors.

The effect of serum albumin on the sensitivity of the CFU-GM to etoposide is both interesting and important. The binding of cytotoxic drugs to protein influences their *in vivo* action but there has not been any previous attempt to evaluate this effect *in vitro* using an assay for myeloid progenitor cells. Etoposide is strongly bound to human serum albumin with ~94% of the drug being bound at typical plasma concentrations (Allen & Craven, 1975). For reasons described in the text serum albumin preparations were present in the assay at a concentration of 25 mg ml$^{-1}$. The contribution made by calf serum amounted to ~6 mg ml$^{-1}$. These combined values are slightly less than normal serum albumin concentration of 34–45 mg ml$^{-1}$. Increasing the albumin concentration further did not affect the response of the progenitor cells to etoposide over the range of drug concentration employed in these experiments. In the absence of added serum albumin the CFU-GM population is sensitive to very low concentrations of etoposide when presumably most of the drug is present in the free form. In the presence of added BSA a ten-fold higher concentration of drug is necessary to elicit a reduction of CFU-GM number although because of the greater slope of the dose response curve the $D_{50}$ concentration was increased only some 4.5 fold. The effect of HSA was even more marked resulting in a decrease of only ~15% at an etoposide concentration of 0.15 $\mu$g ml$^{-1}$.

The binding of etoposide to albumin therefore plays a significant role in the interpretation of the pharmacokinetic data available on etoposide. The increase in $D_{50}$ concentration with HSA agrees well with the protein binding data suggesting ~6% free drug in the plasma. The greater efficacy of HSA compared to BSA agrees with equilibrium dialysis data showing a greater affinity of HSA for etoposide (Littlewood, unpublished data).

These data provide a much clearer picture of the relationship between plasma concentrations of etoposide and the effect on the granulomonocytic progenitor cells. The data can be used as a guide to the plasma levels of drugs which are necessary to achieve a cytotoxic effect on the marrow and secondly as a guide to the maximum plasma levels that can be tolerated prior to reinfusion of bone marrow during autologous bone marrow transplantation.

This work was supported by the Leukaemia Research Appeal for Wales. TJL is in receipt of a grant from the Welsh Office. We would like to thank Bristol Myers (UK) Ltd, for the kind donation of pure etoposide.

### References

ALLEN, L.M. & CREAVEN, P.J. (1975). Comparison of the human pharmacokinetics of VM-26 and VP-16, two antineoplastic epidophyllotoxin glucopyranoside derivatives. *Eur. J. Cancer*, **11**, 697.

BAILEY-WOOD, R., DALLIMORE, C.M. & WHITTAKER, J.A. (1984). Effect of adriamycin on CFU-GM at plasma concentrations found following therapeutic infusions. *Br. J. Cancer*, **50**, 351.

CAVALLI, F., SONNTAG, R.W., JUNG, F., SENN, H.J. & BRUNNER, K.W. (1978). V.P. 16–213 monotherapy for remission induction of small cell lung cancer: A randomized trial using three dosage schedules. *Cancer Treat. Rep.*, **62**, 473.

DREWINKO, B. & BARLOGIE, B. (1976). Survival and cell cycle-progression delay of human lymphona cells *in vitro* exposed to VP16–213. *Cancer Treat. Rep.*, **60**, 1376.

EVANS, W.E., SINLULE, J.A., CROM, W.R., DOW, L.W., LOOK, A.T. & RIVERA, G. (1982). Pharmacokinetics of teniposide (VM-26) and etoposide (VP-16-213) in children with cancer. *Cancer Chemother. Pharmacol.* **7**, 147.

GRIEDER, A., LICHTENSTEIN, N.S. & OLIVERIO, V.T. (1974). Effect of epidophyllotoxin derivative (V.P. 16–213) on macromolecule synthesis and mitosis in mastocytoma cells *in vitro*. *Cancer Res.*, **34**, 1788.

HUANG, C.C., HOV, Y. & WANG, J.J. (1973). Effects of a new anti-tumour agent, epidophyllotoxin, on growth and chromosomes in human haemopoietic cell lines. *Cancer Res.*, **331**, 123.

D'INCALI, M., SESS, C., FARINA, P. & MAGIONI, C. (1981). VP-16 pharmacokinetics after intravenous and oral administration to choriocarcinoma patients. In *New Drugs for Cancer Therapy in the Eighties*. Int. Symp.: Rome, 10–11 April, 1981.

KRISHAN, A., PAIKAK, V. & FREI, E. (1983). Cytofluorimetric studies on the action of podophyllotoxins and epidophyllotoxins (V.M. 26 and VP 16–213) on the cell cycle traverse of human lymphoblasts. *Cancer Res.*, **43**, 1592.

LITTLEWOOD, T.J., SPRAGG, B.P. & BENTLEY, D.P. (1985). When is autologous bone marrow transplantation safe after high dose treatment with etoposide? *Clin. and Lab. Haematol.* (In press).

LOIKE, J.D. & HORWITZ, S.B. (1976). Effects of V.P. 16–213 on the intracellular degradation of DNA in Hela cells. *Biochemistry*, **15**, 5443.

MISRA, N.C. & ROBERTS, D. (1975). Inhibition of 4′ demethylepidophyllotoxin 9-(4,6-0-2-thenlidene-B-D-glucopyranoside) of human lymphoblast cultures in G2 phase of cell cycle. *Cancer Res.*, **35**, 99.

NEWLANDS, E.S. & BAGSHAWE, K.D. (1980). Antitumour activity of the epidophyllin derivative V.P. 16–213 (Etoposide NSC 14150) in gestation choriocarcinoma. *Eur. J. Cancer*, **16**, 401.

RADICE, P.A., BUNN, P.A. & IHDE, D.C. (1979). Therapeutic trials with V.P. 16–213 and V.M. 26; active agents in small cell lung cancer, non Hodgkins lymphomas and malignancies. *Cancer Treat. Rep.*, **63**, 1231.

RIVERA, G., AVERY, T. & ROBERTS, D. (1975). Response of L1210 to combinations of cytosine arabinoside and V.M. 26 and V.P. 16–213. *Eur. J. Cancer*, **11**, 639.

STAHELIN, H. (1970). 4′ demethyl-epidophyllotoxin thenylidene glucoside (VM 26) a podophyllotoxin compound with a new mechanism of action. *Eur. J. Cancer*, **6**, 303.

WOLFF, S.N., FERM, F., McKAY, C.M., HANDE, K.R., HAINSWORT, J.D. & GRECO, F.A. (1983). High dose VP 16–213 and autologous bone marrow transplantation for refractory malignancies-a phase 1 study. *J. Clin. Oncol.*, **11**, 701.

WOZNIAK, A.J. & ROSS, W.E. (1983). DNA damage as a basis for 4′ – demethyl epidodophyllotoxin-9-(4,6-0-ethylidene β-D-glucopyranoside) (etoposide) cytotoxicity. *Cancer Res.*, **43**, 120.

YALOWICH, J.C. & GOLDMAN, I.C. (1984). Analysis of the inhibitory effect of VP 16–213 (epoposide) and podophyllotoxin on thymidine transport and metabolism in Ehlich Ascites tumour cells *in vitro*. *Cancer Res.*, **44**, 984.